# Association of social contact with dementia and cognition: 28-year follow-up of the Whitehall II cohort study

**DOI:** 10.1371/journal.pmed.1002862

**Published:** 2019-08-02

**Authors:** Andrew Sommerlad, Séverine Sabia, Archana Singh-Manoux, Glyn Lewis, Gill Livingston

**Affiliations:** 1 Division of Psychiatry, University College London, London, United Kingdom; 2 Camden and Islington NHS Foundation Trust, London, United Kingdom; 3 Epidemiology of Ageing and Neurodegenerative Diseases, Inserm U1153, Université de Paris, Paris, France; 4 Department of Epidemiology and Public Health, University College London, London, United Kingdom; Johns Hopkins Univeristy, UNITED STATES

## Abstract

**Background:**

There is need to identify targets for preventing or delaying dementia. Social contact is a potential target for clinical and public health studies, but previous observational studies had short follow-up, making findings susceptible to reverse causation bias. We therefore examined the association of social contact with subsequent incident dementia and cognition with 28 years’ follow-up.

**Methods and findings:**

We conducted a retrospective analysis of the Whitehall II longitudinal prospective cohort study of employees of London civil service departments, aged 35–55 at baseline assessment in 1985–1988 and followed to 2017. Social contact was measured six times through a self-report questionnaire about frequency of contact with non-cohabiting relatives and friends. Dementia status was ascertained from three linked clinical and mortality databases, and cognition was assessed five times using tests of verbal memory, verbal fluency, and reasoning. Cox regression models with inverse probability weighting to account for attrition and missingness examined the association between social contact at age 50, 60, and 70 years and subsequent incident dementia. Mixed linear models examined the association of midlife social contact between 45 and 55 years and cognitive trajectory during the subsequent 14 years. Analyses were adjusted for age, sex, ethnicity, socioeconomic status, education, health behaviours, employment status, and marital status. Of 10,308 Whitehall II study participants, 10,228 provided social contact data (mean age 44.9 years [standard deviation (SD) 6.1 years] at baseline; 33.1% female; 89.1% white ethnicity). More frequent social contact at age 60 years was associated with lower dementia risk (hazard ratio [HR] for each SD higher social contact frequency = 0.88 [95% CI 0.79, 0.98], *p* = 0.02); effect size of the association of social contact at 50 or 70 years with dementia was similar (0.92 [95% CI 0.83, 1.02], *p* = 0.13 and 0.91 [95% CI 0.78, 1.06], *p* = 0.23, respectively) but not statistically significant. The association between social contact and incident dementia was driven by contact with friends (HR = 0.90 [95% CI 0.81, 1.00], *p* = 0.05), but no association was found for contact with relatives. More frequent social contact during midlife was associated with better subsequent cognitive trajectory: global cognitive function was 0.07 (95% CI 0.03, 0.11), *p* = 0.002 SDs higher for those with the highest versus lowest tertile of social contact frequency, and this difference was maintained over 14 years follow-up. Results were consistent in a series of post hoc analyses, designed to assess potential biases. A limitation of our study is ascertainment of dementia status from electronic health records rather than in-person assessment of diagnostic status, with the possibility that milder dementia cases were more likely to be missed.

**Conclusions:**

Findings from this study suggest a protective effect of social contact against dementia and that more frequent contact confers higher cognitive reserve, although it is possible that the ability to maintain more social contact may be a marker of cognitive reserve. Future intervention studies should seek to examine whether improving social contact frequency is feasible, acceptable, and efficacious in changing cognitive outcomes.

## Abstract 2

From a longitudinal prospective cohort study, Andrew Sommerlad and colleagues show a protective effect of social contact against developing dementia.

## Author summary

**Why was this study done?:**

- There is a need to identify lifestyle factors that affect the risk of developing dementia so that prevention efforts can be appropriately targeted.
- Previous studies show that having less frequent social contact with others is associated with higher dementia risk, but short follow-up in these studies means that this could be due to social isolation being an early consequence, rather than a cause, of dementia.

**What did the researchers do and find?:**

- We used data from 10,228 participants of the Whitehall II study, aged 45 years on average at the beginning of the study, who were asked on six occasions between 1985 and 2013 about their frequency of social contact with friends and relatives.
- Participants completed cognitive tests administered five times between 1997 and 2016 and were followed through electronic health records until 2017 to ascertain dementia diagnosis.
- We found that greater frequency of social contact at age 60 years was associated with lower risk of developing dementia and that this association was linked to social contact with friends rather than relatives.
- We found that more frequent midlife social contact was associated with higher subsequent cognitive performance, with cognitive differences between those with high and low social contact frequency maintained during average 15 years of cognitive follow-up.

**What do these findings mean?:**

- Our analysis suggests that more frequent social contact during early and midlife may build cognitive reserve, which is maintained and delays or prevents the clinical expression of dementia.
- An alternative explanation for our findings is that early cognitive differences could affect individuals’ subsequent ability to establish and maintain social relationships, as well as increase susceptibility to later dementia.
- A limitation is that our study derived cases of dementia from health records, which has the potential to miss cases of dementia in people who are more socially isolated, which would be likely to underestimate the association between social contact and dementia.

## Introduction

The ageing global population will lead to rising numbers of people with dementia [1], intensifying the need to identify dementia prevention targets. Frequent contact with others has been suggested to be protective [2], either through cognitive reserve increasing resilience to neuropathological damage and delaying dementia onset [3] or encouraging healthier lifestyle behaviours or reduced stress. Meta-analyses of longitudinal studies [4,5] and studies published after these searches were completed for these meta-analyses (since May 2017) [6–8] have reported greater dementia risk and worse cognition [9] in those with less frequent social contact. However seven of the eight studies in the meta-analysis examining dementia had less than 4 years’ follow-up, and 15 of the 19 studies examining cognition had less than 5 years’ follow-up. Because impairments in social function are part of dementia, with increasing dementia severity associated with spending less time with others, and these changes have been described in the prodromal period [10], it is possible that infrequent social contact may in fact be a consequence rather than cause of dementia.

Studies with social contact measures repeated over a long period are needed to establish the direction of the relationship of social contact frequency with dementia and cognitive decline. Furthermore, previous studies have combined social networks of relatives and friends, but it may be that relative contact increases to provide support in the prodromal phase of dementia [11], and cognitive benefit may differ between different social groups, so amalgamating all social contact may obscure associations with dementia. We therefore examined social contact in relation to dementia and cognitive decline in a large longitudinal cohort study with repeated measures of social network contact with friends and relatives over 28 years of follow-up. We hypothesise that higher levels of social contact with both friends and relatives will be associated with subsequent reduced dementia risk and better cognitive function. Our specific aims are to 1) test the association between frequency of social contact with friends and relatives at 50, 60, and 70 years of age and incident dementia; and 2) examine the association between social contact and subsequent cognitive decline.

## Method

### Study design and participants

We undertook a retrospective analsysis of data from the Whitehall II prospective cohort study [12], established in 1985, aiming to study all civil servants aged between 35 and 55 years working in London-based United Kingdom civil service departments; the initial participation rate was 73% [13]. Participants completed questionnaires at each of the 12 waves of data collection and additionally received structured clinical evaluation at 5 yearly intervals during alternate waves. This study is reported as per the Strengthening the Reporting of Observational Studies in Epidemiology (STROBE) guideline [14] (S1 STROBE Checklist).

### Consent and ethical approval

Written informed consent for participation was obtained at each contact. The most recent ethical approval was from the Joint University College London/University College London Hospitals Committee on the Ethics of Human Research (Committee alpha; reference 96/0938).

### Measurements

#### Social network contact

Social network contact was assessed six times (1985–88, 1989–90, 1991–94, 1997–99, 2002–04, 2012–13). Participants completed four ordinal self-rated questions, adapted from the Berkman–Syme social network index [15], about the number and frequency of contact with relatives and friends. Participants were asked 1) ‘do you have any friends or acquaintances you visit or who visit you? (Not necessarily the same person each time)’, 2) ‘how many friends or acquaintances do you see once a month or more?’, 3) ‘are there any relatives outside your household whom you regularly visit or who visit you? (Not necessarily the same person each time)’, and 4) ‘how many relatives do you see once a month or more?’

All questions had five possible response options: ‘Never/almost never’, ‘Once every few months’, ‘About monthly’, ‘About weekly’, and ‘Almost daily’ for questions 1 and 3; ‘None’, 1–2, 3–5, 6–10, and >10 for questions 2 and 4. We generated social contact variables by combining responses from all questions (on a scale of 0–16) and those for friends (0–8) and relatives (0–8). These measures previously showed association with increased mortality risk [15] and worse cognition [16]. Relevant extracts from the questionnaire used in the study are in S1 Text.

#### Dementia

Dementia diagnosis is derived from three comprehensive linked electronic health records through March 2017: NHS Digital’s Hospital Episode Statistics (HES) and Mental Health Services Data (MHDS), which include clinical diagnoses recorded during routine clinical contact in inpatient, outpatient, and community care in the NHS, including memory clinics; and the mortality register [17]. Diagnoses are entered as International Statistical Classification of Diseases and Related Health Problems, Tenth Revision [18] codes with F00x-F03x, F05.1, and G30x-G31.0 indicating dementia of any subtype. HES has sensitivity 78% and specificity 92% for dementia diagnosis, with sensitivity increasing over the past ten years [19], and systematic review data indicate that using multiple different data sources increases sensitivity [20]. The validity of dementia diagnosis using these data sources is demonstrated by the finding of accelerated cognitive decline during the period before dementia diagnosis [21].

#### Cognition

Participants undertook cognitive testing in 1997–99, 2002–04, 2007–09, 2012–13, and 2015–16, measuring three cognitive domains. Verbal fluency was assessed by asking participants to write down as many words beginning with ‘S’ (testing phonemic fluency) and as many animals (semantic fluency) as possible during 1 minute. Short-term verbal memory was assessed by presenting participants with 20 one- or two-syllable words at 2-second intervals, and participants then had 2 minutes to recall in writing as many words as possible. Verbal and mathematical reasoning was assessed using the Alice Heim 4-I test [22]. To reduce measurement error and ease comparison between tests with different score ranges, we standardised all raw test scores to z-scores (mean = 0, standard deviation [SD] = 1) and summed and restandardised scores to yield a global cognitive score, as in previous studies [16].

#### Covariates

We obtained sociodemographic characteristics of participants at baseline: age, sex, ethnicity (white, other ethnicity), and education (no formal education, lower secondary, higher secondary education, graduate, postgraduate). Health behaviours and other characteristics were recorded at all waves: adult socioeconomic status based on grade of last employment (professional, managerial, skilled nonmanual, skilled manual, partly skilled, nonskilled), employment status (employed, not working [unemployed or retired]), and marital status (married, divorced, widowed, single); smoking (never, ex-, or current smoker), alcohol consumption (0, 1–14, >14 alcoholic units/week), and physical activity (hours of moderate or vigorous exercise/week).

### Statistical analysis

Our prospective analysis plan is in S2 Text, and changes to this during the course of our analysis and additional analyses conducted in response to reviewer comments are detailed in S3 Text. We first described the cohort’s sociodemographic characteristics according to dementia status and baseline social contact using *t* test and χ^2^ test. In 1985–88, 2,596 study participants answered question 1 but not 2, for unclear reasons; a full range of question 1 responses was provided by these participants, they were not instructed to skip question 2, and there were no similar missing data at subsequent waves, suggesting that data were missing at random. We imputed values to minimise missing data impact. Because there was moderate correlation between question 1 and 2 responses in wave 1 (Spearman’s rank correlation = 0.54, *p* < 0.001), we imputed mean question 2 response based upon participants’ question 1 response (Question [Q] 1 = 0, Q2 = 0.83; Q1 = 1, Q2 = 1.42; Q1 = 2, Q2 = 1.89; Q1 = 3, Q2 = 2.42; Q1 = 4, Q2 = 2.81).

#### Association between social contact at 50, 60, and 70 years and incident dementia

We examined the association between social contact and subsequent incident dementia. We chose to present results by social contact measurement at age 50, 60, and 70 years, rather than by study wave, to ease interpretation because there was a wide range of ages of study participants. Data on social network contact were extracted for each participant from the wave when they were closest to age 50, 60, and 70 years, allowing a ±5-year margin for each age category, meaning that data from the same study phase were not used at successive age points. After checking the proportionality of hazards assumption, we used Cox regression to model social network’s association (combined friend and relative contact, friend contact only, relative contact only) with incident dementia using age as timescale. We censored participants at the date of dementia diagnosis, death, or 31st March 2017, whichever came first. We had no a priori hypothesis derived from previous literature to suggest need to analyse by subgroups; therefore, we did not do so.

Results are presented as hazard ratios (HRs) for dementia according to one SD increase in social contact and adjusted for birth cohort (using 5-year categories) and sex, and then with the addition of ethnicity, education, socioeconomic status; smoking status, physical activity, and alcohol consumption (health behaviours); employment status; and finally, marital status. Sex, ethnicity, and education are taken from baseline, and other covariates are taken from time of exposure measurement. Missing covariates are imputed from adjacent waves, if available.

The analyses of social network at age 50, 60, and 70 years and subsequent dementia were based on 8,483, 7,348, and 4,870 participants, respectively, because of nonparticipation and missing data. Missingness, either by nonparticipation or missing social contact or cognitive data, was associated with demographic characteristics, social network contact, and incident dementia (S1, S2 and S3 Tables). We therefore used inverse probability weighting [23] so that analyses reflected surviving baseline study participants using the inverse of the probability of inclusion in fully adjusted models, using data on sociodemographic and behavioural factors, social network contact, dementia status, and the interaction between social contact and dementia in study participants who were alive at each age point. Unweighted results are presented in appendices.

#### Association between social contact and cognitive function

We used mixed linear models [24] with random intercept and slope to examine the association between mean social contact and subsequent cognitive trajectories. We used the mean of social contact during four waves over approximately 10 years (1985–88, 1989–90, 1991–94, and 1997–99) to reduce measurement error and characterise social contact over a prolonged period of time and divided respondents into tertiles of mean social contact of approximately equal size. We examined the mean rate of change in standardised z-scores for global cognitive score and individual cognitive tests, using age in years divided by 10 as the timescale, centred at age 56 years (mean age in 1997–99), meaning that coefficients are presented as number of SD change per 10 years.

We undertook analyses of the association of low, medium, and high mean social contact during 1985–88 to 1997–99 and cognitive trajectories from 1997–99 to 2015–16, with results presented adjusted for sex, age, and age squared to represent the accelerated cognitive decline at older ages, and then for the covariates described above (birth cohort, ethnicity, education, socioeconomic status, health behaviours, employment, and marital status) as recorded in 1997–99.

#### Post hoc analyses

We conducted several post hoc analyses in response to peer review comments. We repeated analyses of the association of social contact frequency at different age points, as outlined above, with the addition of cognitive status as a covariate, using the global cognitive z-score at the time of exposure measurement; we only conducted this analysis at age 60 and 70 years because of missing cognition data at age 50 years. We conducted another analysis with additional adjustment for chronic physical illness at time of exposure measurement (body mass index as a continuous variable, hypertension [defined as either taking an antihypertensive or having systolic blood pressure ≥141 mmHg], diabetes mellitus [defined as either having previously received diagnosis of diabetes mellitus, taking antidiabetic medication, having fasting plasma glucose ≥7.1 mmol/L, or plasma glucose 2 hours after oral glucose tolerance test ≥11.1 mmol/L], and coronary heart disease [derived from HES]). In another post hoc analysis, we repeated our primary analysis using Cox regression to examine the association between social contact frequency at age 50, 60, and 70 years and incident dementia but imposed a 3-year washout period whereby we excluded study participants who had less than 3 years follow-up because of death, incident dementia, or end of follow-up.

To examine in more detail whether reverse causation underlies associations between social contact and dementia, we examined whether change in social contact from age 60 to 70 years—generated by subtracting social contact score at 60 years from score at 70 years so that a positive value indicated more social contact—was associated with incident dementia using Cox regression, censored at date of dementia diagnosis, death, or 31st March 2017, whichever came first. Analyses were adjusted sequentially for birth cohort (using 5-year categories) and sex; ethnicity, education, and socioeconomic status; smoking status, physical activity, and alcohol consumption (health behaviours); employment; and marital status, all measured at age 70 years and adjusted for social contact frequency at age 60 years. Inverse probability weighting was used to weight analyses for the probability of participants being included in these models. We also generated categories of social change from tertiles of social network contact at age 60 years and 70 years: remain low, remain medium, remain high, increasing, decreasing (full details on categorisation in S9 Table). We then calculated the association between these five categories—with ‘remain high’ as the reference group—and incident dementia, with adjusted and inverse probability weighted as above, using covariates measured at 70 years.

Because previous studies have suggested differences in the associations of lifestyle factors and cognitive trajectories between people who did and did not subsequently develop dementia [25,26], we repeated analyses stratified by dementia status. We also examined for interaction with age in the association between social contact and cognitive performance at baseline and cognitive decline, using time, in years centred on the 1997–99 phase, as timescale and social contact as a continuous variable.

All analyses were undertaken using STATA SE version 14; 2-sided *p* < 0.05 was considered statistically significant.

## Results

**Fig 1:**
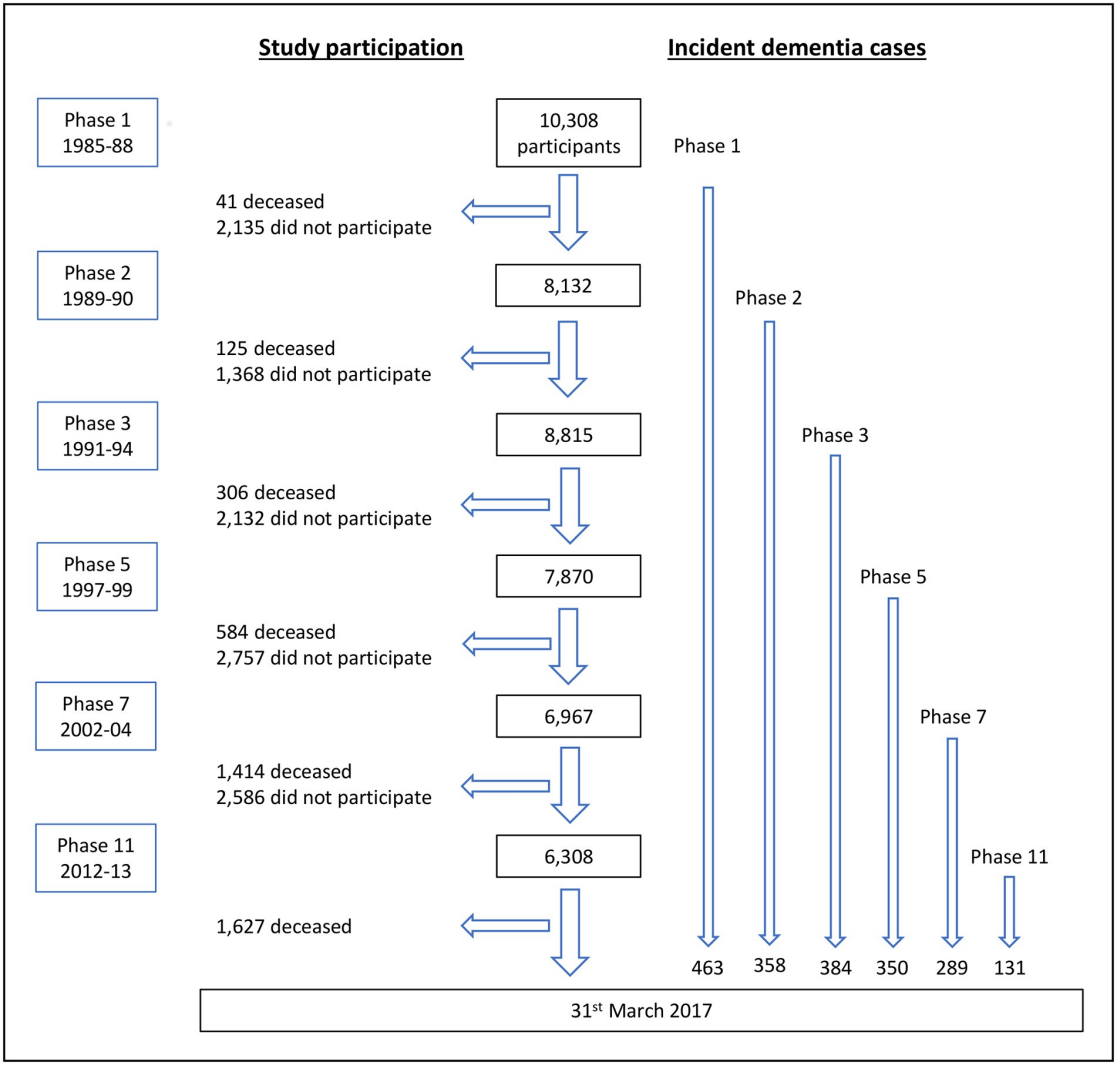
Flow chart of study participants.

Table 1 shows demographic characteristics: 10,308 people participated initially, of whom 10,228 provided social contact data. Fig 1 summarises participant flow: 1,627 participants had died and 463 developed dementia by the end of follow-up over a mean 28.6 years (SD 4.9, maximum 31.8 years). The mean age at dementia diagnosis was 75.9 (SD 5.6, range 56.9–86.0). In univariate analyses, dementia status was associated with sex, age, marital status, ethnicity, education, alcohol consumption, smoking, physical activity, and social contact. Social contact was associated with sex, socioeconomic class, alcohol consumption, and physical activity.

**Table 1:** Baseline demographics of study participants according to dementia status (n = 10,308).

Characteristic		All Participants n = 10,308		No Dementia n = 9,845		Dementia n = 463		*p*-value	Mean Social Contact Score (SD) n = 10,228	*p*-Value
		n	%	n	%	n	%			
**Sex**	Male	6,895	66.9	6,635	67.4	260	56.2	*p* < 0.001	6.9 (2.7)	*p* < 0.001
	Female	3,413	33.1	3,210	32.6	203	43.8		7.2 (2.8)	
**Age**	Mean (SD)	44.9 (6.1)		44.7 (6.0)		50.2 (4.7)		*p* < 0.001		*p* = 0.08
	Min, max	34.1, 56.3		34.1, 56.3		35.2, 56.0				
**Marital status**	Married	7,608	73.8	7,285	74.0	323	69.8	*p* < 0.001	7.0 (2.7)	*p* = 0.06
	Single	1,690	16.4	1,613	16.4	77	16.6		6.8 (2.8)	
	Divorced	833	8.1	782	7.9	51	11.0		6.9 (2.9)	
	Widowed	139	1.4	129	1.3	10	2.2		6.9 (2.6)	
	Missing	38	0.4	36	0.4	2	0.4		6.5 (3.2)	
**Ethnicity**	White	9,181	89.1	8,787	89.3	394	85.1	*p* = 0.005	7.0 (2.7)	*p* = 0.57
	Other	1,127	10.9	1,058	10.8	69	14.9		6.9 (2.9)	
**Social class**	Professional	1,133	11.0	1,086	11.0	47	10.2	*p* < 0.001	7.2 (2.5)	*p* = 0.003
	Managerial	1,895	18.4	1,828	18.6	67	14.5		7.0 (2.6)	
	Skilled nonmanual	1,426	13.8	1,379	14.0	47	10.2		6.9 (2.7)	
	Skilled manual	1,976	19.2	1,920	19.5	56	12.1		6.9 (2.7)	
	Partly skilled	1,541	15.0	1,473	15.0	68	14.7		6.8 (2.8)	
	Nonskilled	2,337	22.7	2,159	21.9	178	38.4		7.0 (3.0)	
Age leaving education	No qualifications	1,029	10.0	953	9.7	76	16.4	*p* < 0.001	7.1 (2.9)	*p* = 0.39
	Lower secondary	3,870	37.5	3,666	37.2	204	44.1		7.0 (2.9)	
	Higher secondary	2,745	26.6	2,653	27.0	92	19.9		6.9 (2.7)	
	Graduate	2,097	20.3	2,030	20.6	67	14.5		7.0 (2.6)	
	Postgraduate	567	5.5	543	5.5	24	5.2		6.8 (2.5)	
Alcohol (units/wk)	0	1,873	18.2	1,745	17.7	128	27.7	*p* < 0.001	6.6 (3.1)	*p* < 0.001
	1–7	3,882	37.7	3,695	37.5	187	40.4		7.0 (2.7)	
	8–14	2,040	19.8	1,983	20.1	57	12.3		7.1 (2.6)	
	>14	2,419	23.5	2,334	23.7	85	18.4		7.1 (2.7)	
	Missing	94	0.9	88	0.9	6	1.3		7.0 (2.5)	
Smoking	Never smoked	5,069	49.2	4,844	49.2	225	48.6	*p* < 0.001	6.9 (2.7)	*p* = 0.06
	Ex-smoker	3,281	31.8	3,147	32.0	134	28.9		7.0 (2.7)	
	Current smoker	1,886	18.3	1,787	18.2	99	21.4		7.1 (2.8)	
	Missing	72	0.7	67	0.7	5	1.1		7.3 (2.6)	
Physical activity (hours/wk)	Median (IQ range)	3 (1, 5)		3 (1,5)		2 (0, 5)		*p* < 0.001		*p* < 0.001
	Min, max	0, 70		0, 70		0, 25				
	Missing	158		145		13				
All social contact score	Mean (SD)	7.0 (0.03)		7.0 (0.03)		6.7 (0.1)		*p* = 0.02	N/A	
	Min, max	0, 16		0, 16		0, 14				
	Missing	494		465		29				

**Abbreviations**: GHQ-30, General Health Questionnaire-30; IQ range, interquartile range; N/A, not applicable; SD, standard deviation; wk, week.

### Association between social network contact at 50, 60, and 70 years and incident dementia

**Fig 2:**
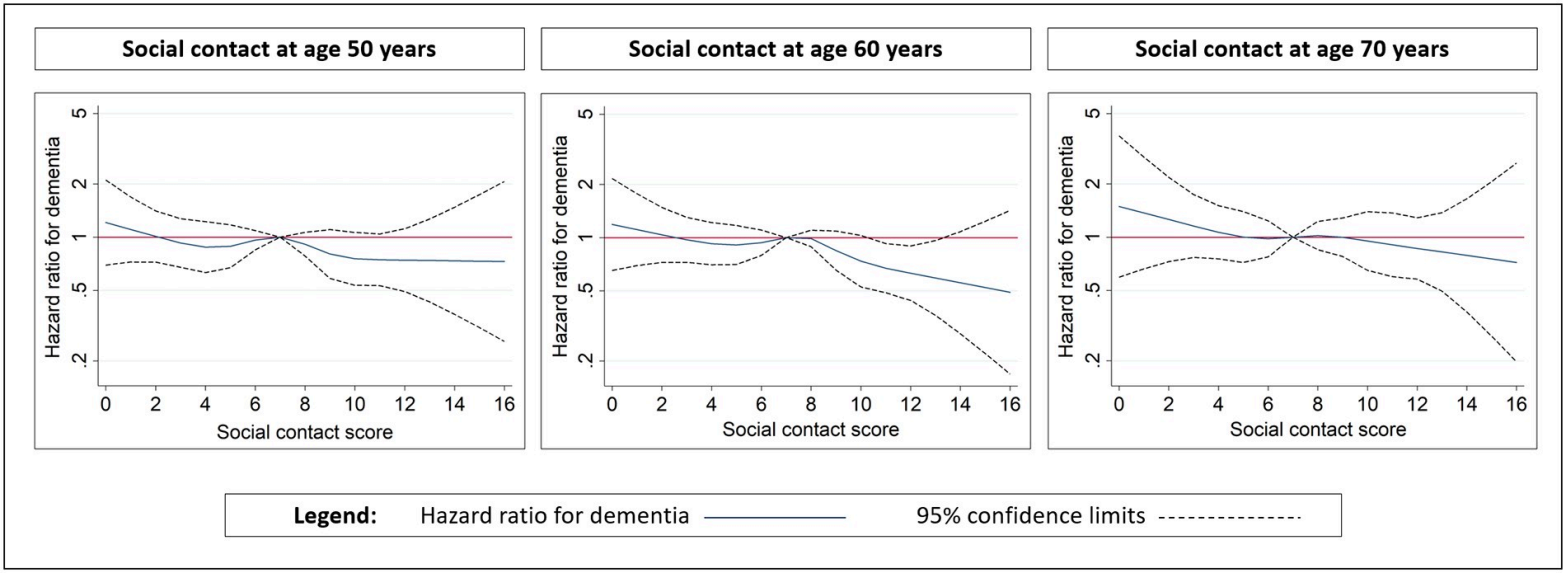
Association of frequency of social contact with friends and relatives at age 50, 60 and 70 years and incident dementia: Plot of HR for dementia according to social contact score. Weighted Cox regression models adjusted for age, sex, education, social class, ethnicity, smoking, alcohol, exercise, employment status, and marital status. Reference for social contact is score 7 (mean score at baseline). HR, hazard ratio.

Social network contact increased from age 50 to 60 to 70 years (total social network score 6.9, 7.5, 8.1, respectively), with the most change from increasing contact with friends and acquaintances (from 3.9 at 50 to 4.7 at 70) and some increase in contact with relatives (increased from 3.0 to 3.4) (S4 Table). In adjusted and weighted models, a higher amount of social contact at 60 years was associated with reduced risk of dementia (HR for one SD increase in social contact = 0.88, 95% CI 0.79, 0.98, *p* = 0.02) (Table 2). Point estimates of the association of social contact at age 50 years and 70 years and dementia were similar (HR 0.92 [0.83, 1.02], *p* = 0.13 and 0.91 [0.78, 1.06], *p* = 0.23 respectively) but not statistically significant. Higher contact with friends at age 60 years was associated with lower risk of dementia (HR = 0.90 [0.81, 1.00], *p* = 0.05), but associations were not found for contact with friends at other age points. There was no evidence that social contact with relatives was associated with dementia. HR for dementia associated with each social contact score, with 7 as reference value, is shown in Fig 2.

**Table 2:** Association between social network contact at different ages and subsequent incident dementia: HR for dementia associated with higher levels of social network contact.

**Age**			**50 years**	**60 years**	**70 years**
**Mean years follow-up (SD)**			**23.1 (6.2)**	**14.6 (6.9)**	**7.5 (4.4)**
**Number included in fully adjusted model (weighted n)**			**8,487 (10,278)**	**7,439 (10,141)**	**4,888 (9,237)**
**Number of incident dementia cases in those who participated**			**362**	**351**	**221**
**All social contact**	**Adjusted for age and sex**	Per SD increase in social contact	0.90 (0.81, 1.00)	**0.86 (0.77, 0.95)**	0.87 (0.75, 1.01)
	**+ education, social class, and ethnicity**		0.91 (0.82, 1.01)	**0.88 (0.79, 0.98)**	0.89 (0.77, 1.04)
	**+ smoking, alcohol, and exercise**		0.92 (0.83, 1.02)	**0.88 (0.79, 0.98)**	0.91 (0.78, 1.06)
	**+ employment status**		0.92 (0.83, 1.02)	**0.88 (0.79, 0.98)**	0.91 (0.78, 1.06)
	**+ marital status**		0.92 (0.83, 1.02)	**0.88 (0.79, 0.98)**	0.91 (0.78, 1.06)
**n included in fully adjusted model (weighted n)**			**8,643 (10,279)**	**7,617 (10,141)**	**5,035 (9,236)**
**Friend contact**	**Adjusted for age and sex**	Per SD increase in social contact	0.92 (0.83, 1.03)	**0.86 (0.78, 0.96)**	**0.86 (0.76, 0.99)**
	**+ education, social class, and ethnicity**		0.95 (0.85, 1.05)	**0.90 (0.80, 1.00)**	0.89 (0.77, 1.02)
	**+ smoking, alcohol, and exercise**		0.96 (0.86, 1.07)	**0.90 (0.81, 1.00)**	0.91 (0.79, 1.05)
	**+ employment status**		0.96 (0.86, 1.07)	0.90 (0.81, 1.00)	0.92 (0.80, 1.05)
	**+ marital status**		0.96 (0.86, 1.07)	**0.90 (0.81, 1.00)**	0.91 (0.80, 1.05)
**n included in fully adjusted model (weighted n)**			**8,493 (10,278)**	**7,449 (10,141)**	**4,889 (9,240)**
**Relative contact**	**Adjusted for age and sex**	Per SD increase in social contact	0.91 (0.81, 1.01)	0.92 (0.83, 1.03)	0.93 (0.80, 1.08)
	**+ education, social class, and ethnicity**		0.90 (0.81, 1.00)	0.92 (0.83, 1.03)	0.94 (0.80, 1.09)
	**+ smoking, alcohol, and exercise**		0.91 (0.82, 1.01)	0.92 (0.83, 1.03)	0.94 (0.80, 1.10)
	**+ employment status**		0.91 (0.82, 1.01)	0.92 (0.83, 1.03)	0.94 (0.81, 1.11)
	**+ marital status**		0.91 (0.82, 1.02)	0.92 (0.83, 1.03)	0.94 (0.80, 1.11)

Weighted according to inverse of probability of inclusion in fully adjusted model; bold results indicate *p* < 0.05. **Abbreviations**: HR, hazard ratio; SD, standard deviation.

In sensitivity analyses without inverse probability weighting (S5 Table), results were similar at age 50 and 60 years, and the association between social contact and dementia was underestimated at age 70 (unweighted = 0.95 [0.83, 1.09], *p* = 0.49 versus weighted 0.91 [0.78, 1.06], *p* = 0.23). Associations were also similar when we additionally adjusted our analyses for baseline cognitive ability (S6 Table): there was loss of statistical power because of the smaller number of participants who had these data, and the HR for dementia associated with one SD higher in all social contact at age 60 was 0.87 (0.72, 1.04), *p* = 0.13, compared to 0.88 (0.79, 0.98), *p* = 0.02 in analyses not adjusted for cognitive function. Our post hoc analyses with additional adjustment for chronic physical illness (S7 Table) also gave similar results to our primary analyses: the HRs for dementia in those with more frequent social contact at 50, 60, and 70, respectively, were 0.91 (0.82, 1.01), *p* = 0.09; 0.87 (0.78, 0.97), *p* = 0.01; and 0.90 (0.77, 1.05), *p* = 0.18. Using a 3-year washout period (S8 Table) had little effect on the estimates at age 50 (HR 0.92 [0.83, 1.02], *p* = 0.13) or 60 (HR 0.89 [0.80, 0.99], *p* = 0.03), but the HR for dementia associated with social contact frequency at age 70 years, with 143 participants excluded from the analysis, was 1.01 (0.87, 1.18), *p* = 0.88, compared to 0.91 (0.78, 1.06), *p* = 0.23 in models without a washout period.

We found no association between change in social network score from age 60 to 70 years and incident dementia (S9 Table), with mean follow-up from age 70 years of 7.6 years. One point increase in all social contact from 60 to 70 years was not associated with dementia (HR 1.00 [0.94, 1.06], *p* = 0.99). Compared to participants whose social contact remained high, no other category of social change was associated with a significantly higher risk of dementia.

### Association between social network contact and subsequent cognitive decline

Cognition was assessed in 7,540 participants, who had mean 3.8 assessments over 14.3 (SD 5.6, max 19.4) years; scores are in S10 Table. Mean cognitive decline was 0.40 (0.40, 0.41) SDs per 10 years. In adjusted mixed linear models examining the association of social contact frequency and cognition (Table 3), higher mean social contact during 1985–88 to 1997–99 was associated with higher cognition; high versus low social contact tertile had 0.07 (0.03, 0.11) SDs higher (*p* = 0.002) combined cognitive score, and this difference was driven by contact with friends but not contact with relatives, for which there were no cognitive differences. Social contact with friends and relatives was not associated with rate of cognitive decline (cognitive change per 10 years in high versus low social contact = −0.01 SD [−0.03, 0.01], *p* = 0.49), meaning that baseline differences were maintained over time. However, slightly faster cognitive decline was found in those with high frequency of contact with friends (−0.03 SDs [−0.05, −0.00], *p* = 0.02). Full results from the adjusted models for the low, medium, and high tertiles are in S11 Table, showing a gradient across the three social contact tertiles.

**Table 3:** Differences in baseline cognition and cognitive change according to preceding social contact frequency.

		Difference, in SDs, in Baseline Cognition for Those with High versus Low Social Contact (95% CI)				Difference, in SDs, in Cognitive Change per 10 Years for Those with High versus Low Social Contact (95% CI)			
		Global Cognition	Verbal Fluency	Verbal Memory	Reasoning	Global Cognition	Verbal Fluency	Verbal Memory	Reasoning
**All social contact**	**Adjusted for age and sex**	**0.09 (0.04, 0.14)**	**0.10 (0.05, 0.15)**	**0.05 (0.00, 0.10)**	0.03 (−0.03, 0.08)	−0.00 (−0.03, 0.02)	0.00 (−0.02, 0.03)	0.00 (−0.03, 0.03)	−0.00 (−0.02, 0.02)
	**Fully adjusted**	**0.07 (0.03, 0.11)**	**0.08 (0.03, 0.12)**	**0.05 (0.00, 0.10)**	0.01 (−0.03, 0.05)	−0.01 (−0.03, 0.01)	−0.00 (−0.03, 0.02)	−0.01 (−0.04, 0.02)	−0.01 (−0.03, 0.01)
**Friend contact**	**Adjusted for age and sex**	**0.22 (0.17, 0.28)**	**0.22 (0.17, 0.27)**	**0.10 (0.05, 0.15)**	**0.17 (0.12, 0.22)**	−0.02 (−0.05, 0.00)	−0.02 (−0.05, 0.01)	−0.02 (−0.05, 0.02)	−0.00 (−0.03, 0.02)
	**Fully adjusted**	**0.08 (0.03, 0.12)**	**0.10 (0.05, 0.15)**	**0.04 (0.01, 0.09)**	0.02 (−0.02, 0.06)	**−0.03 (−0.05, −0.00)**	−0.02 (−0.05, 0.00)	−0.02 (-0.06, 0.01)	−0.01 (−0.03, 0.01)
**Relative contact**	**Adjusted for age and sex**	**−0.13 (−0.19, −0.07)**	**−0.10 (−0.16, −0.04)**	**−0.08 (−0.14, −0.03)**	**−0.17 (−0.23, −0.11)**	0.01 (−0.02, 0.03)	0.01 (−0.02, 0.04)	0.03 (−0.01, 0.06)	0.00 (−0.02, 0.02)
	**Fully adjusted**	0.01 (−0.03, 0.06)	0.02 (−0.03, 0.07)	−0.01 (−0.06, 0.05)	0.00 (−0.04, 0.05)	0.00 (−0.02, 0.03)	0.00 (−0.03, 0.03)	0.02 (−0.02, 0.05)	−0.00 (−0.03, 0.02)

Baseline cognition centred at age 56 years; number included in analysis for combined cognition = 7,092, for verbal fluency and verbal memory = 7,120, for reasoning = 7,132; fully adjusted model adjusted for age, sex, education, social class, ethnicity, smoking, alcohol, exercise, employment status, and marital status at baseline; bold figures indicate *p* < 0.05. **Abbreviations**: SD, standard deviation.

In analyses stratified by dementia status (S12 Table), mean cognitive decline for those who did (n = 298) and did not (n = 7,253) develop dementia was 0.95 (0.88, 1.03) and 0.39 (0.38, 0.40) SD per 10 years. Baseline cognitive differences according to preceding frequency of social contact were more pronounced in those who subsequently developed dementia than in those who did not (dementia cases: baseline global cognition was 0.42 [0.06, 0.75] SD higher [*p* = 0.02] for those with high than low social contact; dementia-free 0.06 [0.02, 0.10], *p* = 0.006). We found no evidence for interaction with age in the association between social contact and cognitive performance at baseline (*p* = 0.11) or cognitive decline (*p* = 0.34).

## Discussion

In this analysis of a large prospective study with 28 years’ follow-up, we found more frequent social network contact at age 60 but not at age 50 or 70 years was associated with reduced risk of dementia, and this association was driven by contact with friends rather than relatives. We also found that more frequent social contact during 10 years from mean age 45 to 55 was associated with a higher level of cognition but not with rate of subsequent cognitive change; this association was related to social contact with friends but not with relatives. Though the associations between social contact and dementia incidence are of borderline statistical significance and associations were not found at all age points, these findings taken together suggest that having more frequent social contact during late middle age might reduce dementia risk independently of social and lifestyle factors.

The association between social contact frequency and dementia and cognition may be related to social contact directly improving cognition and reducing dementia risk, and the existence of such a causal relationship is supported by several findings. The associations between social contact and subsequent incident dementia were similar in size at age 50, 60, and 70 years, although they were only statistically significant at age 60; the nonrobust findings using social contact at 50 and 70 years indicate the need for further research. Associations between social contact and dementia and cognition followed a dose–response relationship. Reverse causation bias is unlikely because measurements of social contact preceded assessment for dementia by mean 15 years between assessment at age 60 years and the end of the follow-up, which is likely to be beyond the time at which prodromal changes of dementia could feasibly lead to reverse causation. Furthermore, in our analysis of the association of social contact change with dementia risk, there was no evidence that individuals whose social contact decreased between age 60 and 70 were at higher risk of incident dementia, as we might expect if associations were related to prodromal dementia changes. However, reverse causation remains a potential explanation for our findings. It is plausible that higher childhood and adolescent cognitive ability allows individuals to develop and maintain more frequent social contact, meaning that social contact may partially mediate the association between cognitive reserve and subsequent dementia risk. Future interventional studies are required to clarify the casual relationship.

There are several plausible mechanisms by which social contact could reduce dementia risk: more social contact could build cognitive reserve by exercising cognitive domains such as memory and language, thereby delaying dementia onset. A postmortem study of 89 people found that higher levels of monthly social contact at mean 3 years before death modified the relationship between neuropathology and cognition such that neuropathology load was less strongly associated with cognitive decline in people with more frequent social contacts [27], consistent with the concept of cognitive reserve.

The present findings that contact with friends, but not relatives, was associated with better cognitive outcomes may indicate that greater cognitive effort is involved in keeping in contact with friends compared to relatives, thereby building cognitive reserve. Alternatively, contact with friends could theoretically lead to greater enjoyment and lower stress [28] because friends reflect individual choices. Another potential explanation for the differences in associations between friend and relative contact is that the number of relatives is usually limited, whereas the number of potential friendships is theoretically unlimited. For example, those with high cognition and low risk of dementia may have no relatives but many friends, which would strengthen the association for friends but not relatives. In addition, the scale used in our study has a potential ceiling effect for relatives, but not friends, because a participant with maximum frequency (daily) contact with their only available relative could only score 5 out of 8 on the relative subscale, possibly resulting in underestimation of the association between frequency of contact with relatives and cognition and dementia.

Our finding of association between more frequent midlife social contact and better cognitive function but not subsequent cognitive change is consistent with previous research on cognitive reserve [29–32] and offers support to the hypothesis that social contact builds cognitive reserve. The higher baseline cognition and slightly faster cognitive decline in people with high, compared to low, social contact with friends, particularly in those who went on to develop dementia, is consistent with previous studies that have found these cognitive trajectories associated with markers of cognitive reserve, such as education and occupational status, in people who subsequently developed dementia [25,26]. This finding suggests that dementia-related neuropathology may partly attenuate the cognitive reserve benefits conferred by social contact.

There are alternative plausible mechanisms for a beneficial effect of social contact on dementia risk. Social contact could affect subsequent health behaviours such that socially active individuals have healthier diets, drink less alcohol, or take more exercise, although in univariate analyses of baseline data, we found that smokers and those who drank alcohol had slightly higher mean social contact than nonsmokers and alcohol abstainers, and risk estimates were similar before and after adjustment for these factors. Social contact could also affect dementia risk through the effect of stress; less social contact is associated with elevated cortisol response, a detrimental effect of stress on hippocampal networks has been demonstrated in animal models [33], and persistent midlife stress has been associated with elevated dementia risk in epidemiological studies [34].

The magnitude of effect in this study was smaller than previously reported. The pooled relative risk estimates from the recent meta-analyses [4,5] of the association between higher social contact frequency and dementia risk (inverted to compare high versus low social contact) were 0.64. Our figures indicate a 12% reduction in dementia incidence for each SD higher social contact score at age 60 years, equivalent to, for example, the difference between seeing 1–2 friends every few months compared to almost daily. A study with up to 4 years’ follow-up reported risk of dementia for those having daily, compared to less than weekly, contact to be 43% lower (adjusted for age, education, health, and baseline cognition) [35]. Having daily, compared to no, contact with relatives and friends in a Swedish study with 3 years’ follow-up was associated with 29% reduced incidence of dementia (age, sex, and education adjusted) [36]. Our lower estimate may indicate overestimation in previous studies because of reverse causation bias due to short follow-up and insufficient adjustment for confounders or underestimation in our study. In our post hoc analysis using a 3-year washout period, results at age 50 and 60 were similar, suggesting that the associations at those age points were not due to some participants having very short follow-up. However, the association between social contact at age 70 years and subsequent dementia was attenuated when we excluded 143 study participants with less than 3 years follow-up, which indicates the need for long follow-up or a washout period when considering dementia risk related to domains that are susceptible to prodromal change.

### Strengths and limitations

To our knowledge, our study has longer follow-up than any previous research, and we assessed social contact on multiple occasions, reducing measurement error and allowing us to exclude reverse causation more confidently than previously possible. Attrition was low, with 80%–90% of those surveyed participating at each successive study wave, and use of inverse probability weighting allowed direct comparison between results from exposure measurement at different ages. This study has limitations, however. Social contact was self-reported and thus subject to reporting bias. However, our long follow-up meant that we were unlikely to include measurements of social contact during dementia prodrome. We aimed to control for confounders, but there may be unmeasured confounders, such as hearing impairment, for which we could not adjust our analyses, and our covariates were treated as constant over time because attrition over the follow-up did not allow use of time-varying covariates, potentially affecting the magnitude of associations.

Questionnaire-derived data lack detail because we have no information about the nature and quality of contact between study participants, such as conversational activity and how cognitively stimulating or enjoyable the social contact may have been, potentially resulting in lower power to detect association. Our ascertainment of dementia status using electronic health records, rather than standardised clinical assessment of all study participants, is a potential source of bias. The data sources we used include most diagnosed dementia, but national diagnosis rates are currently around 68% [37], and unmarried people have been shown to be less likely to receive dementia diagnoses [19,38]. This may extend to less socially active people who lack an informant to recognise emerging dementia symptoms and encourage clinic attendance, thereby underestimating the association between social contact and incident dementia. However, electronic health records reduce the risk of attrition bias when needing face-to-face examination of participants.

Study participants were all working in the UK civil service, possibly limiting generalisability, although UK employment trends mean that similar to Whitehall participants, there are few manual workers and jobs are predominantly office-based and technology-focused [39]. Additionally, study participants were relatively young to develop dementia, and young-onset dementias have a relatively larger genetic contribution [40], meaning that lifestyle factors’ influence is smaller, so our findings may underestimate the risk of low social contact in the whole population. Testing the replicability of these findings in other cohorts may increase the generalisability.

### Clinical implications and future research

There is need to identify possible intervention targets to prevent dementia, and our study suggests around a 10% reduction in dementia risk per SD increase in social contact in late middle age, so this may be a target for intervention. Future observational studies with long follow-up duration should aim to replicate these findings. Considering the general health benefits associated with good-quality social relationships [41] and the lack of known adverse effects, people at risk of developing dementia should be encouraged to increase social contact. Potential public health approaches to reducing older people’s isolation and increasing societal connectedness may be beneficial. One recent feasibility study examined the effect of daily internet-based conversational interactions on cognitive decline in people with mild cognitive impairment, finding better verbal fluency at 12-week follow-up in those who received the intervention compared to the control group [42], and future studies should examine the feasibility of increasing social contact and its effect on cognition and dementia risk with longer follow-up. Although our study did not specifically examine this period, considering the general health benefits and potential for effect on cognition, there may be value in conducting intervention trials in preclinical dementia when loss of social contact may be occurring as part of the disease and perpetuating disease progression. Clinicians can advise people with mild cognitive impairment and early dementia to engage in socially stimulating activities.

### Conclusions

In this observational cohort study with 28 years’ follow-up, we found that more frequent social contact during mid–late life was associated with lower risk of dementia over 28 years’ follow-up and a better cognitive trajectory during the subsequent 15 years. This association could be attributed to social contact with friends, rather than with relatives. These findings may suggest that more frequent social contact during early and midlife builds a cognitive reserve that is maintained and confers later protection, although an alternative explanation is that higher early cognitive ability allows individuals to establish and maintain social relationships and that the greater cognitive ability protects against subsequent dementia. Replication of this study in other cohort settings and future intervention studies should explore this relationship in more detail.

## Supporting information

S1 STROBE Checklist Strengthening the Reporting of Observational Studies in Epidemiology (STROBE) checklist. (DOCX)

S1 Text Excerpt from Whitehall II phase 1 questionnaire regarding social contact frequency. (DOCX)

S2 Text Social engagement and risk of dementia and cognitive decline: Analysis plan. (DOCX)

S3 Text Timeline of data analysis. (DOCX)

S1 Table Association of baseline characteristics of Whitehall II participants and association with participation at successive age points. (DOCX)

S2 Table Association of baseline characteristics of Whitehall II participants and association with missing social data at successive age points. (DOCX)

S3 Table Association of characteristics of Whitehall II participants with missing cognitive data in successive study phases. (DOCX)

S4 Table Description of social contact at age points. (DOCX)

S5 Table Association between social network contact and subsequent incident dementia: Weighted and unweighted HR for dementia associated with higher levels of social network contact. HR, hazard ratio.
(DOCX)

S6 Table Association between social network contact at different ages and subsequent incident dementia, with additional adjustment for baseline cognitive ability: HR for dementia associated with higher levels of social network contact. HR, hazard ratio.
(DOCX)

S7 Table Association between social network contact at different ages and subsequent incident dementia, with additional adjustment for baseline chronic physical illness: HR for dementia associated with higher levels of social network contact. HR, hazard ratio.
(DOCX)

S8 Table Association between social network contact at different ages and subsequent incident dementia, with 3-year washout period: HR for dementia associated with higher levels of social network contact. HR, hazard ratio.
(DOCX)

S9 Table Association between social contact change from age 60 to 70 years and subsequent incident dementia during mean 7.5 years’ follow-up: HR for dementia associated with continuous and categorical social contact change. HR, hazard ratio.
(DOCX)

S10 Table Description of cognitive function test scores at each study phase. (DOCX)

S11 Table Differences in baseline cognition and cognitive change per 10 years between Whitehall II study participants with preceding medium and high social contact frequency, compared to those with low social contact. (DOCX)

S12 Table Differences in baseline cognition and cognitive change per 10 years between Whitehall II participants with preceding high and low social contact frequency, according to whether they subsequently developed dementia. (DOCX)

## Abbreviations

GHQ-30: General Health Questionnaire-30
HES: Hospital Episode Statistics
HR: hazard ratio
IQ range: interquartile range
MHDS: Mental Health Services Data
SD: standard deviation
STROBE: Strengthening the Reporting of Observational Studies in Epidemiology
