# Supplementary material for: Association of social contact with dementia and cognition: 28-year follow-up of the Whitehall II cohort study
Source: PLoS Med. 2019 Aug 2;16(8):e1002862. doi: 10.1371/journal.pmed.1002862 (PMC6677303; doi:10.1371/journal.pmed.1002862)
Supplement: S1 Text — (DOCX) [file pmed.1002862.s002.docx]

**Supplementary text 1: Excerpt from Whitehall II phase 1 questionnaire regarding social contact frequency**


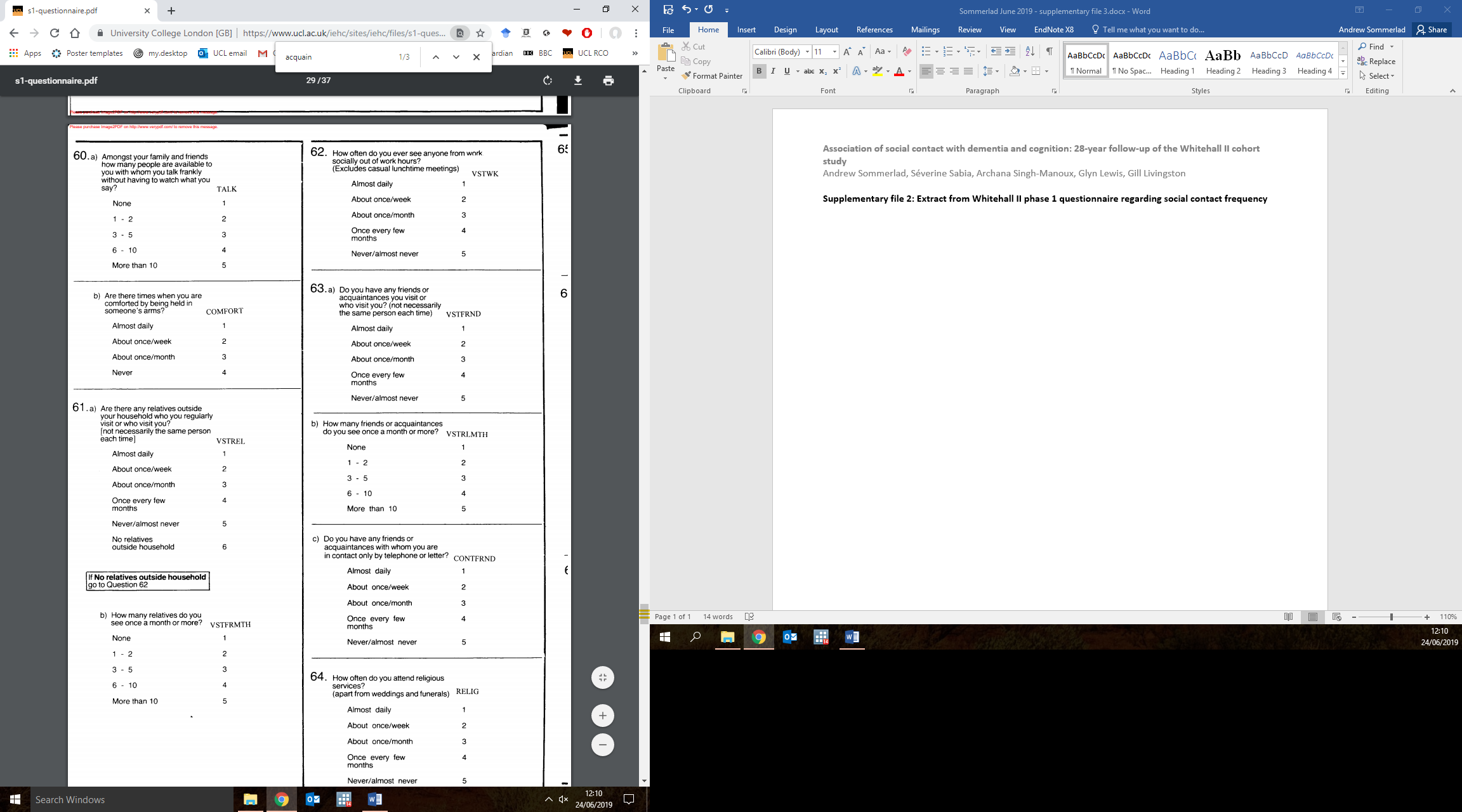


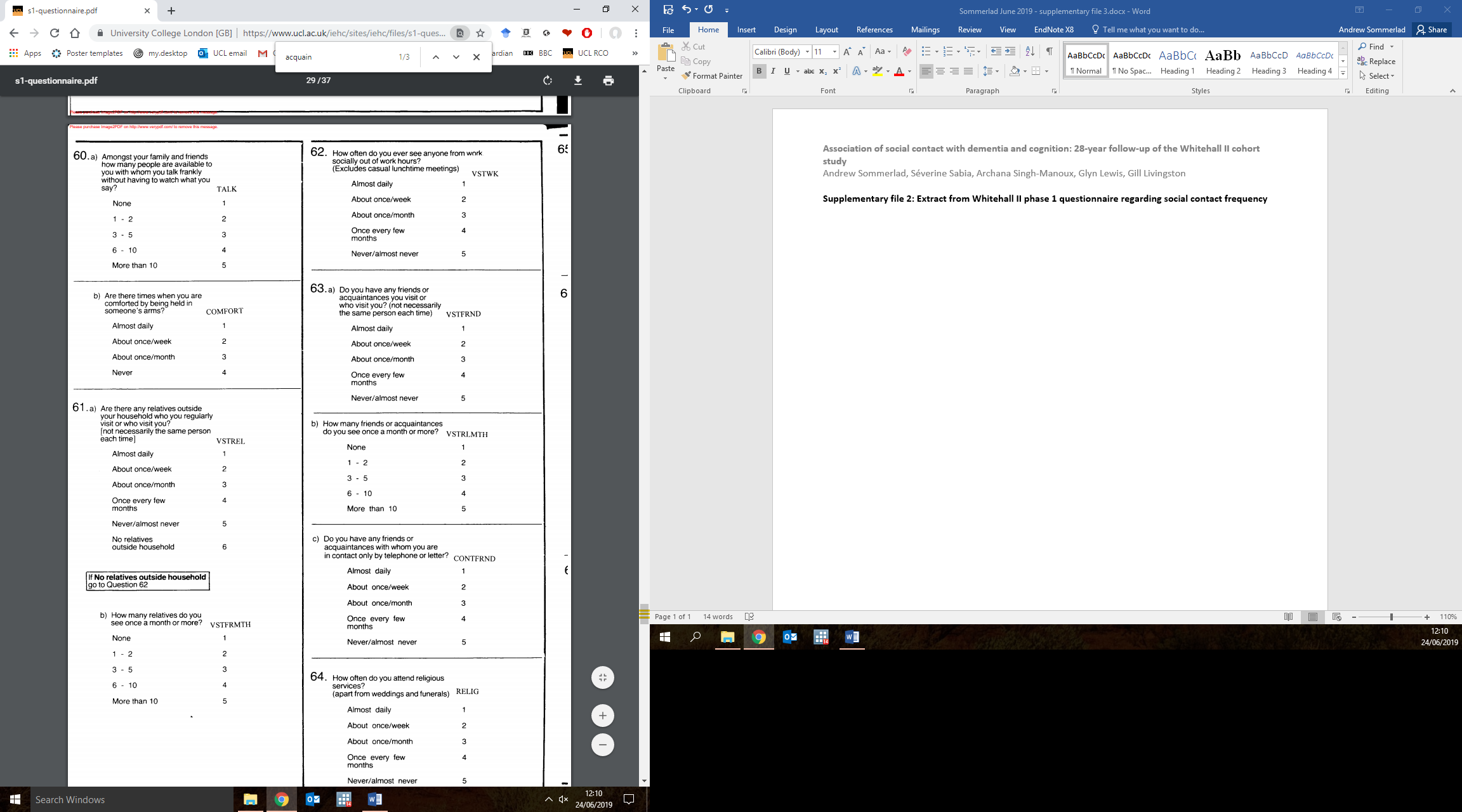


**Notes:** Full data collection questionnaires are available from the Whitehall II website: <https://www.ucl.ac.uk/iehc/research/epidemiology-and-public-health/research/whitehall-ii/data-collection>
