# Supplementary material for: Association of social contact with dementia and cognition: 28-year follow-up of the Whitehall II cohort study
Source: PLoS Med. 2019 Aug 2;16(8):e1002862. doi: 10.1371/journal.pmed.1002862 (PMC6677303; doi:10.1371/journal.pmed.1002862)
Supplement: S2 Text — (DOCX) [file pmed.1002862.s003.docx]

**Supplementary text 2: Social engagement and risk of dementia and cognitive decline: Analysis plan (June 2018)**

# Objectives:

1. Test association between social engagement and incident dementia
2. Model social engagement trajectories prior to development of dementia
3. Association between change in social engagement and incident dementia
4. Examine association between social network contact and subsequent cognitive decline

# Method

### Design:

Prospective study of longitudinal data that explores the association between social engagement, as the primary exposure variable, and cognitive decline or dementia, as the outcome, in a population of people observed at multiple waves of data collection.

### Participants:

This study is based on data from the Whitehall II study (1) (WII), a large occupational study, established in 1985, with a target population of all London-based civil servants aged between 35 and 55 years, which has collected social, biological and clinical data during 12 phases over 30 years. Eligible participants included all men and women aged between 35 and 55 years working within the London offices of 20 departments of the UK civil service. Ethical approval has been granted by the London-Harrow Research Ethics Committee and the Scotland Research Ethics Committee.

### Measurements

WII collects data during visits to the research clinic at 5-yearly intervals. Data is also collected by questionnaire between research visits. Table 1 summarises the data collection schedule.

**Table 1: Summary of Whitehall II study data collection schedule and participant numbers**

|  |  |  |  | **Social measures (n)** | | **Cognitive measures (n)** | | |  |
| --- | --- | --- | --- | --- | --- | --- | --- | --- | --- |
| Phase | Year | Participants n (%) | Mean Age (y) | Social network contact | Leisure activity participation | Exec func’ verbal mem etc | MMSE | Dementia ascertainment (HES, MHDS, Mortality data) | |
| **1** | 1985-8 | 10308 | 44.9 | 9839 |  |  |  |  | |
| 2 |  |  |  |  |  |  |  |  | |
| **3** | 1991-4 | 8815 (86.6) | 50.3 | 8301 |  | 3500 |  |  | |
| 4 |  |  |  |  |  |  |  |  | |
| **5** | 1997-9 | 7870 (78.7) | 56.0 | 6691 | 7000 | 6030 | 1615 |  | |
| 6 |  |  |  |  |  |  |  |  | |
| **7** | 2002-4 | 6967 (71.6) | 61.2 | 6542 | 6660 | 6362 | 6372 |  | |
| 8 |  |  |  |  |  |  |  |  | |
| **9** | 2007-9 | 6761 (72.3) | 66.0 |  | 6540 | 6071 | 6141 |  | |
| 10 |  |  |  |  |  |  |  |  | |
| **11** | 2012-3 | 6318 (70.9) | 71 |  | 6540 |  |  |  | |
| **12** | 2016 | In progress |  |  |  |  |  | ~ 463 | |

#### Outcome variables

##### Dementia

Dementia diagnosis data is derived from linked routine clinical data sources; Hospital episode statistics, mental health dataset and mortality data.

###### Hospital episode statistics:

Diagnostic data are from records of general (nonpsychiatric) inpatient admissions to any hospital in England and the clinical diagnoses recorded on each hospital discharge summary by the treating clinical team. Diagnoses are recorded as International Statistical Classification of Diseases and Related Health Problems, 10th Revision codes and each admission has up to 20 diagnostic codes. The method of admission (elective or nonelective) is also recorded. Diagnoses recorded in HES are those clinically identified during the admission, obtained from correspondence with primary care, or derived from pre-existing clinical records such as previous hospital medical records—some record systems prepopulate diagnosis fields with previously recorded chronic conditions. Dementia diagnostic status also obtained from MHDS and mortality data.

##### Cognition

Cognitive measures were introduced as a pilot testing scheme in WII phase 3 and implemented throughout the cohort in phase 5. The cognitive measures are sensitive to cognitive change (2). Previous studies have combined individual test scores to create a global cognitive score to minimise the effect of individual test’s measurement error (3). I will use this as my primary outcome, but will also examine specific cognitive domains:

###### Memory

Short-term verbal memory

###### Executive function

Abstract non-verbal reasoning (AH4 test)

Verbal fluency (s-words and animal fluency)

###### Combined cognitive test

Mini-mental state examination (4)

####

#### Exposure variables:

Social network contact

Four ordinal self-rated questions from questionnaire about number and frequency of contact with relatives and friends (*Figure 1*), assessed on six occasions (*table 1*). Continuous social network contact variables generated from combining responses from all questions (0-17); relative questions (0-9); and friend questions (0-8).

**Figure 1: Social engagement**

| **Relatives** | **1)** How often do you regularly visit or are visited by these relatives? | No relatives/Never/almost never; Once every few months; About monthly; About weekly; Almost daily | 0-4 |
| --- | --- | --- | --- |
|  | **2)** How many relatives do you see once a month or more? | No relatives; 1-2; 3-5; 6-10; >10 | 0-4 |
| **Friends** | **3)** Do you have any friends or acquaintances you visit or who visit you? (Not necessarily the same person each time) | Never/almost never; Once every few months; About monthly; About weekly; Almost daily | 0-4 |
|  | **4)** How many friends or acquaintances do you see once a month or more? | None; 1-2; 3-5; 6-10; >10 | 0-4 |
|  | | **0-16** | |

During phase 1 and 2, one additional question was asked to participants about socialising with work colleagues: (‘How often do you ever see anyone from work socially out of work hours? (Excludes casual Iunch-time meetings)), with scoring 0 to 4 (Never/almost never; Once every few months; About monthly; About weekly; Almost daily).

##### Marital status

#### Covariates

- Gender
- Age
- Ethnicity
- Education (self-reported)
- Adult socioeconomic status, based on last held employment grade
- Life events
  - Marital status (at entry to study?)
  - Employment status
  - Bereavement
- Health behaviours
  - Smoking status
  - Alcohol consumption
  - Frequency of participation in moderate exercise
- Marital status has been recorded at each wave of data collection, and details of transition between marital states (widowed, divorced, separated) including year of change, have been recorded.

# Statistical analysis:

#### Descriptive analyses

Describe the basic characteristics of the cohort including duration between assessments and participation:

##### Social engagement

- Frequency of contact with social network at phase 1, 2, 3, 5, 7 and 11
- Change in contact with social network between phase 3 to 5, 5 to 7
- Change in contact with social network between phase 5 to 7, 7 to 9, 9 to 11

I will analyse the consistency of social change between successive phases of data collection

##### Cognitive function

- Cognitive function at phase 5 and subsequent phases
- Change in cognitive function between phase 5 to 7, 7 to 9 etc
- Number of cases of dementia

##### Covariates

I will describe cohort characteristics in terms of covariate measurements.

#### Exploratory data analyses

I will conduct exploratory data analyses to assess missingness bias, potential for confounding, and bivariate relationships.

For these analyses, I will use chi-square analyses for categorical variables, and independent samples *t* tests and ANOVA for continuous variables.

##### Missingness

To assess missingness bias associated with missing exposure variables, I will examine whether subjects with missing or incomplete data on social engagement differ from subjects with complete social engagement histories on any of the covariates or on cognitive performance or decline.

After conducting these analyses, we will exclude subjects with missing or incomplete social engagement data from further exploratory analyses and models.

To assess missingness bias associated with death or withdrawal from the study, we will conduct exploratory investigations to examine whether subjects who died or dropped out of the study before phase 5 differed on baseline social engagement, social disengagement, covariates and on rate o0066 cognitive decline

##### Confounders

To assess potential for confounding and describe bivariate relationships, I will analyse whether potential covariates relate to social engagement and social disengagement (*in particular those who have retired or been bereaved change in social engagement*); and whether social engagement, change in social engagement and covariates and moderators relate to cognitive decline.

#### Social network contact

##### Association between social network contact and incident dementia


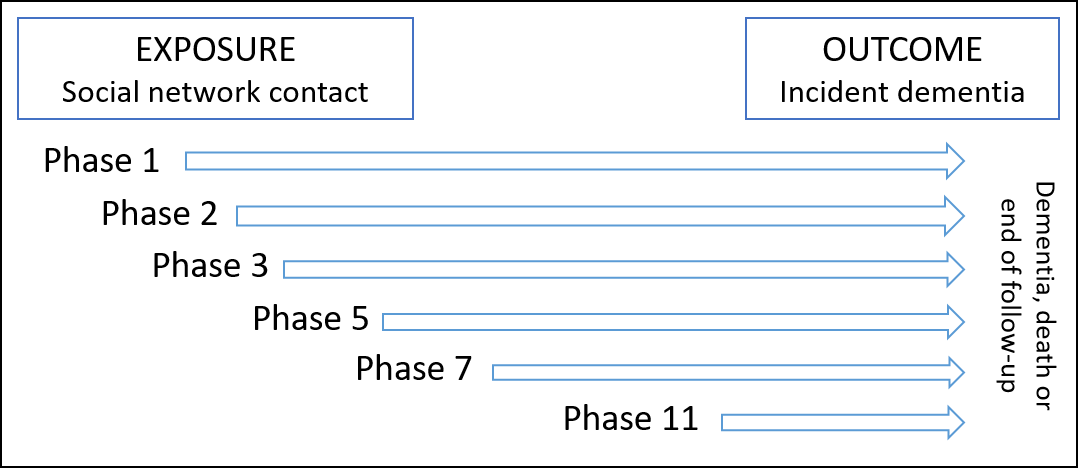


Cox regression using exposure variables from successive study phases (i.e. diminishing duration of follow-up), censored at date of dementia diagnosis, death, or 31^st^ March 2017.

Outcome is incident dementia

Exposures are all social contact; contact with friends; contact with relatives. Analyses undertaken with exposure variables as continuous and categorical (grouped into tertiles based on distribution of values at phase 1).

All results are presented as unadjusted; adjusted for age and sex; additionally adjusted for ethnicity, education, socioeconomic status and health behaviours; additionally adjusted for employment and marital status. Age, sex, ethnicity, education and socioeconomic status are taken from phase 1; health behaviours, employment and marital status covariates are taken from time of exposure measurement. Any missing covariates are imputed from the previous phase if available.

Sensitivity analysis of association between incident dementia and all social contact and friend contact summed with contact with work colleague variable for phase 1 and 2.

##### Social network contact trajectories prior to development of dementia

Mixed linear models with backward timescale

##### Association between change in social network contact and incident dementia


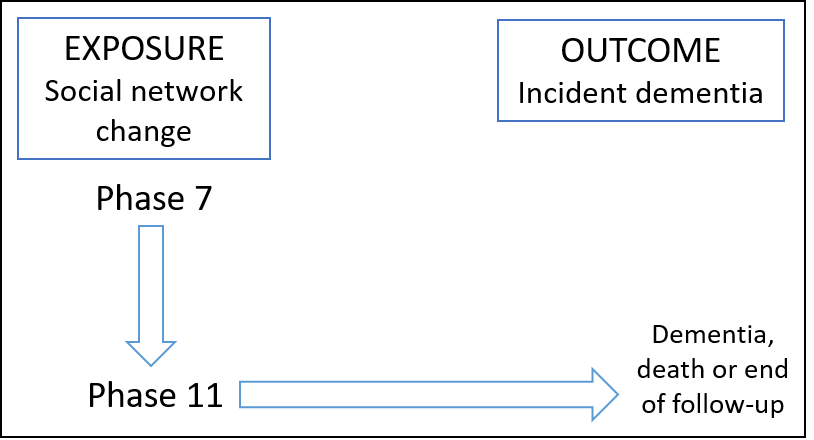


Generated continuous exposure variables of social network contact change during 9 years between phase 7 and 11 (positive value = increase in social network contact)

Cox regression using exposure variable, censored at date of dementia diagnosis, death, or 31^st^ March 2017.

Outcome is incident dementia

Exposures are all social contact; contact with friends; contact with relatives.

All results are presented as unadjusted; adjusted for age and sex; additionally adjusted for ethnicity, education, socioeconomic status and health behaviours; additionally adjusted for employment and marital status. Age, sex, ethnicity, education and socioeconomic status are taken from phase 1; health behaviours, employment and marital status covariates are taken from time of exposure measurement. Any missing covariates are imputed from the previous phase if available.

##### Association between social network contact and subsequent cognitive decline


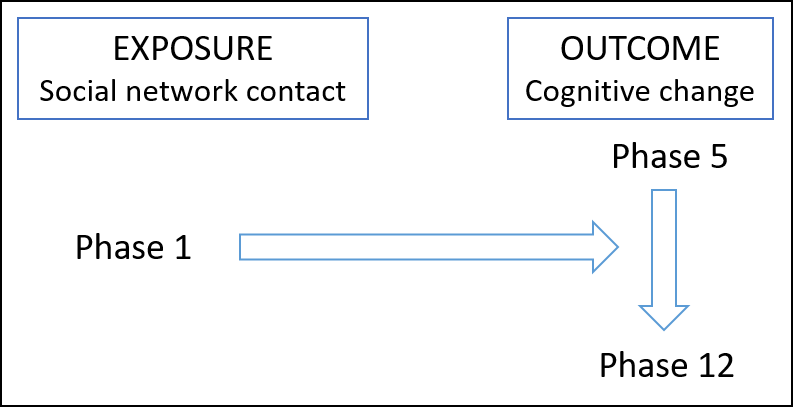


Mixed linear models with random intercept and random slope fitted, testing association between social network contact at phase 1 and cognitive function change between phase 5 and 12 (five phases of cognitive function testing).

Outcome data is cognitive function. Each cognitive test transformed into a z score (difference between value and mean / standard deviation (based on mean and sd from first cognitive testing phase)). Global cognitive function variables generated by summing the other cognitive test z scores and again repeating z-score process.

Exposures are all social contact; contact with friends; contact with relatives. Analyses undertaken with exposure variables as continuous and categorical (grouped into tertiles based on distribution of values at phase 1).

All results are presented as unadjusted; adjusted for age and sex; additionally adjusted for ethnicity, education, socioeconomic status and health behaviours; additionally adjusted for employment and marital status. Covariates are taken from phase 1.
