# Supplementary material for: Association of social contact with dementia and cognition: 28-year follow-up of the Whitehall II cohort study
Source: PLoS Med. 2019 Aug 2;16(8):e1002862. doi: 10.1371/journal.pmed.1002862 (PMC6677303; doi:10.1371/journal.pmed.1002862)
Supplement: S3 Text — (DOCX) [file pmed.1002862.s004.docx]

Supplementary text 3: Timeline of data analysis

March 2017: Data from phase 12 of study available for analysis

March 2018: Data from linkage to electronic health records until 31/03/2017 retrieved and processed.

June 2018: Analysis plan (appendix 2) written.

June 2018 – January 2019: Statistical analysis undertaken.

Changes to a priori plan during analysis:

1. To aid interpretation of results, we decided to present results by age of exposure, rather than study phase at exposure measurement. We generated variables for exposure and covariate data at age 50, 60, and 70 years, allowing a margin of +/- 5 years, by extracting the data from the phase closest to these ages. The 5 year margin meant that data from the same study phase was not used at successive age points.
2. Our exploration of the association of social contact, dementia and cognition with missing data and non-participation in the study indicated potential risk of attrition and missingness bias, so we decided to use inverse probability weighting, as used in previous studies by our group, to weight participants according to probability of inclusion in fully adjusted models.
3. Due to the structure of data collection, we found unreliable results when we attempted to model trajectories of social contact prior to dementia development using mixed linear models with backward trajectory (objective 2). Measurement of social contact had been measured irregularly, including with a 10 year gap between phase 7 and 11, meaning that modelling social contact changes between those phases was unreliable. We therefore did not proceed with this analysis and instead plan to conduct a future study with another cohort with regular measurements of social contact frequency.
4. For objective 4, we chose to generate an exposure variable reflecting mean social contact frequency during phases 1 to 5, rather than assessing social contact frequency only at phase 1, as we judged that this would minimise missing social contact data and reflect social contact frequency over a longer period of time.
5. Following review of other studies of cognitive change and dementia, which had found differing cognitive trajectories according to dementia status, we undertook a post-hoc sensitivity analysis of the association of social contact frequency with cognitive trajectories, stratified by dementia status.

April 2019: Submission to PLOS Medicine

May 2019: In response to PLOS Medicine reviewers, we undertook additional post hoc analyses:

1. We repeated analyses of the association of social contact frequency at different age points, as outlined above, with the addition of cognitive status as a covariate, using the global cognitive z-score at the time of exposure measurement; we only conducted this analysis at age 60 and 70 years, due to missing cognition data at age 50 years.
2. We conducted another analysis with additional adjustment for chronic physical illness at time of exposure measurement (body mass index as a continuous variable, hypertension (defined as either taking an antihypertensive or having systolic blood pressure ≥141mmHg), diabetes mellitus (defined as either having previously received diagnosis of diabetes mellitus, taking anti-diabetic medication, having fasting plasma glucose ≥7.1mmol/L, or plasma glucose 2 hours after oral glucose tolerance test ≥11.1mmol/L), and coronary heart disease (derived from hospital episode statistics)).
3. In another post-hoc analysis, we repeated our primary analysis using cox regression to examine the association between social contact frequency at age 50, 60, and 70 years and incident dementia but imposed a 3 year washout period whereby we excluded study participants who had less than 3 years follow-up, either due to death, incident dementia, or end of follow-up.
4. We examined whether change in social contact from age 60 to 70 years – generated by subtracting social contact score at 60 years from score at 70 years so a positive value indicated more social contact – was associated with incident dementia using Cox regression, censored at date of dementia diagnosis, death, or 31^st^ March 2017, whichever came first. Analyses were adjusted sequentially for birth cohort (using 5 year categories) and sex; ethnicity, education and socioeconomic status; smoking status, physical activity, and alcohol consumption (health behaviours); employment; and marital status, all measured at age 70 years, and adjusted for social contact frequency at age 60 years. Inverse probability weighting was used to weight analyses for the probability of participants being included in these models. We also generated categories of social change from tertiles of social network contact at age 60 years and 70 years; remain low, remain medium, remain high, increasing, decreasing (full details on categorisation in appendix). We then calculated the association between these five categories, with ‘remain high’ as reference group, and incident dementia, adjusted and inverse probability weighted as above, using covariates measured at 70 years.
