# Supplementary material for: Association of social contact with dementia and cognition: 28-year follow-up of the Whitehall II cohort study
Source: PLoS Med. 2019 Aug 2;16(8):e1002862. doi: 10.1371/journal.pmed.1002862 (PMC6677303; doi:10.1371/journal.pmed.1002862)
Supplement: S1 Table — (DOCX) [file pmed.1002862.s005.docx]

Supplementary table 1: Association of baseline characteristics of Whitehall II participants and association with participation at successive age points

|  | 50 years (n=8,853) | | 60 years (n=7,710) | | 70 years (n=5,137) | |
| --- | --- | --- | --- | --- | --- | --- |
| Participated? | **Yes** | **No** | **Yes** | **No** | **Yes** | **No** |
| N | **8,853** | **1,455** | **7,710** | **2,598** | **5,137** | **5,171** |
| Mean baseline age | 45.2 | 43.1 | 45.5 | 43.2 | 47.1 | 42.8 |
| p value | < 0.001 | | < 0.001 | | <0.001 | |
| % Male | 67.3 | 64.2 | 68.6 | 61.8 | 69.8 | 64.0 |
| p value | 0.02 | | <0.001 | | <0.001 | |
| % Married at baseline | 74.7 | 70.5 | 75.9 | 68.6 | 77.7 | 70.5 |
| p value | 0.001 | | <0.001 | | <0.001 | |
| Mean baseline social network score | 7.0 | 6.8 | 7.0 | 6.7 | 7.0 | 6.9 |
| p value | 0.03 | | < 0.001 | | 0.02 | |
| % Dementia case | 4.4 | 4.9 | 4.8 | 3.7 | 4.7 | 4.3 |
| p value | 0.44 | | 0.02 | | 0.28 | |
