# Supplementary material for: Association of social contact with dementia and cognition: 28-year follow-up of the Whitehall II cohort study
Source: PLoS Med. 2019 Aug 2;16(8):e1002862. doi: 10.1371/journal.pmed.1002862 (PMC6677303; doi:10.1371/journal.pmed.1002862)
Supplement: S2 Table — (DOCX) [file pmed.1002862.s006.docx]

Supplementary table 2: Association of baseline characteristics of Whitehall II participants and association with missing social data at successive age points

|  | 50 years (n=8,853) | | 60 years (n=7,710) | | 70 years (n=5,137) | |
| --- | --- | --- | --- | --- | --- | --- |
| Complete data? | **Yes** | **No** | **Yes** | **No** | **Yes** | **No** |
| n | **8,622** | **231** | **7,476** | **234** | **4,950** | **187** |
| Mean baseline age | 45.2 | 45.5 | 45.5 | 45.0 | 47.1 | 47.3 |
| p value | 0.43 | | 0.20 | | 0.52 | |
| % Male | 67.4 | 64.1 | 68.7 | 65.8 | 70.0 | 62.6 |
| p value | 0.28 | | 0.35 | | 0.03 | |
| % Married at baseline | 74.7 | 71.9 | 76.3 | 63.3 | 78.2 | 65.2 |
| p value | 0.32 | | < 0.001 | | < 0.001 | |
| Mean baseline social network score | 7.0 | 4.3 | 7.1 | 5.5 | 7.1 | 6.2 |
| p value | < 0.001 | | < 0.001 | | < 0.001 | |
| % Dementia case | 4.3 | 9.5 | 4.7 | 6.8 | 4.6 | 7.5 |
| p value | < 0.001 | | 0.13 | | 0.07 | |
