# Supplementary material for: Association of social contact with dementia and cognition: 28-year follow-up of the Whitehall II cohort study
Source: PLoS Med. 2019 Aug 2;16(8):e1002862. doi: 10.1371/journal.pmed.1002862 (PMC6677303; doi:10.1371/journal.pmed.1002862)
Supplement: S3 Table — (DOCX) [file pmed.1002862.s007.docx]

Supplementary table 3: Association of characteristics of Whitehall II participants with missing cognitive data in successive study phases

| Study phase | 5 (n=7,780) | | 7 (n=6,967) | | 9 (n=6,761) | | 11 (n=6,308) | | 12 (n=5,631) | | 5-12 (n=8,355) ^b^ | |
| --- | --- | --- | --- | --- | --- | --- | --- | --- | --- | --- | --- | --- |
| Complete data? | **Yes** | **No** | **Yes** | **No** | **Yes** | **No** | **Yes** | **No** | **Yes** | **No** | **Yes** | **No** |
| n | **5,882** | **1,988** | **5,984** | **653** | **6,009** | **752** | **5,486** | **822** | **4,734** | **897** | **7,551** | **804** |
| Mean baseline age | 44.6 | 45.4 | 44.5 | 46.4 | 44.2 | 46.5 | 43.8 | 46.3 | 43.4 | 45.6 | 44.6 | 46.0 |
| p value | < 0.001 | | < 0.001 | | < 0.001 | | < 0.001 | | < 0.001 | | < 0.001 | |
| % Male | 71.3 | 64.3 | 71.4 | 59.4 | 72.1 | 57.1 | 72.5 | 58.9 | 73.6 | 61.1 | 70.3 | 59.3 |
| p value | < 0.001 | | < 0.001 | | < 0.001 | | < 0.001 | | < 0.001 | | < 0.001 | |
| % Married at baseline | 75.9 | 73.8 | 76.4 | 73.2 | 76.5 | 72.7 | 76.7 | 75.1 | 77.4 | 74.1 | 75.9 | 69.2 |
| p value | 0.07 | | 0.07 | | 0.02 | | 0.29 | | 0.04 | | < 0.001 | |
| Mean social network score ^a^ | 7.4 | 7.2 | 7.6 | 7.3 | 7.6 | 7.6 | 8.0 | 7.6 | 8.1 | 7.7 | 7.0 | 6.8 |
| p value | 0.04 | | 0.12 | | 0.88 | | 0.002 | | 0.001 | | 0.04 | |
| % Dementia case | 3.8 | 6.3 | 3.8 | 7.5 | 2.8 | 7.4 | 1.8 | 6.8 | 0.9 | 4.5 | 4.0 | 7.8 |
| p value | < 0.001 | | < 0.001 | | < 0.001 | | < 0.001 | | < 0.001 | | < 0.001 | |

Notes: ^a^ Social network score taken from respective study phase, apart from phase 9 and 12, when social contact was not measured, when score from preceding study phase was used. ^b^ Column refers to participants who have provided cognitive function data at any point during study phases 5 to 12
