# Supplementary material for: Association of social contact with dementia and cognition: 28-year follow-up of the Whitehall II cohort study
Source: PLoS Med. 2019 Aug 2;16(8):e1002862. doi: 10.1371/journal.pmed.1002862 (PMC6677303; doi:10.1371/journal.pmed.1002862)
Supplement: S4 Table — (DOCX) [file pmed.1002862.s008.docx]

Supplementary table 4: Description of social contact at age points

| Age  (n) |  | Social contact scale | | Friend subscale | | Relative subscale | |
| --- | --- | --- | --- | --- | --- | --- | --- |
|  |  | **n** | **%** | **n** | **%** | **n** | **%** |
| 50 years  (9,178) | Mean (SD)  Range | 6.9 (2.8)  0, 16 | | 3.9 (1.9)  0, 8 | | 3.0 (1.8)  0, 8 | |
|  | *Missing* | 491 | 5.3 | 325 | 3.5 | 485 | 5.3 |
| 60 years  (8,060) | Mean (SD)  Range | 7.5 (3.0)  0, 16 | | 4.3 (2.0)  0, 8 | | 3.2 (1.9)  0, 8 | |
|  | *Missing* | 533 | 6.6 | 350 | 4.3 | 523 | 6.5 |
| 70 years  (5,320) | Mean (SD)  Range | 8.1 (3.1)  0, 16 | | 4.7 (2.0)  0, 8 | | 3.4 (2.0)  0, 8 | |
|  | *Missing* | 336 | 6.3 | 183 | 3.4 | 335 | 6.3 |
|  |  |  | |  | |  | |
