# Supplementary material for: Association of social contact with dementia and cognition: 28-year follow-up of the Whitehall II cohort study
Source: PLoS Med. 2019 Aug 2;16(8):e1002862. doi: 10.1371/journal.pmed.1002862 (PMC6677303; doi:10.1371/journal.pmed.1002862)
Supplement: S5 Table — HR, hazard ratio. (DOCX) [file pmed.1002862.s009.docx]

Supplementary table 5: Association between social network contact and subsequent incident dementia: Weighted and unweighted hazard ratio for dementia associated with higher levels of social network contact

| Age: | | | 50 years | 60 years | 70 years |
| --- | --- | --- | --- | --- | --- |
| Mean years follow-up | | | **23.1 (6.2)** | **14.6 (6.9)** | **7.5 (4.4)** |
| n included | | | **8,487** | **7,439** | **4,888** |
| All social contact | **Weighted** | Per standard deviation increase in social contact | 0.92 (0.83, 1.02) | **0.88 (0.79, 0.98)** | 0.91 (0.78, 1.06) |
|  | **Unweighted** |  | 0.91 (0.82, 1.01) | **0.88 (0.79, 0.98)** | 0.95 (0.83, 1.09) |
|  | | |  |  |  |
| n included | | | **8,643** | **7,617** | **5,035** |
| Friend contact | **Weighted** | Per standard deviation increase in social contact | 0.96 (0.86, 1.07) | **0.90 (0.81, 1.00)** | 0.91 (0.80, 1.05) |
|  | **Unweighted** |  | 0.96 (0.86, 1.06) | **0.90 (0.80, 1.00)** | 0.93 (0.81, 1.06) |
|  | | |  |  |  |
| n included | | | **8,493** | **7,449** | **4,889** |
| Relative contact | **Weighted** | Per standard deviation increase in social contact | 0.91 (0.82, 1.02) | 0.92 (0.83, 1.03) | 0.94 (0.80, 1.11) |
|  | **Unweighted** |  | **0.89 (0.80, 0.99)** | 0.92 (0.83, 1.03) | 0.99 (0.86, 1.13) |

Notes: Weighted using inverse probability weighting for inclusion in fully-adjusted model; all results adjusted for age, sex, education, social class, ethnicity, smoking, alcohol, exercise, employment status and marital status; Bold results indicate p<0.05; Social class based upon occupational grade
