# Supplementary material for: Association of social contact with dementia and cognition: 28-year follow-up of the Whitehall II cohort study
Source: PLoS Med. 2019 Aug 2;16(8):e1002862. doi: 10.1371/journal.pmed.1002862 (PMC6677303; doi:10.1371/journal.pmed.1002862)
Supplement: S7 Table — HR, hazard ratio. (DOCX) [file pmed.1002862.s011.docx]

Supplementary table 7: Association between social network contact at different ages and subsequent incident dementia, with additional adjustment for baseline chronic physical illness: hazard ratio for dementia associated with higher levels of social network contact

| Age | | 50 years | 60 years | 70 years |
| --- | --- | --- | --- | --- |
| Mean years f/u | | **23.1 (6.2)** | **14.6 (6.9)** | **7.5 (4.4)** |
| Number included in fully adjusted model | | **8,458 (10,242)** | **7,326 (9,978)** | **4,736 (8,975)** |
| Number of incident dementia cases in those who participated | | **362** | **363** | **221** |
| All social contact | Hazard ratio for dementia per standard deviation higher social contact | 0.91 (0.82, 1.01)  p=0.09 | 0.87 (0.78, 0.97)  p=0.01 | 0.90 (0.77, 1.05)  p=0.18 |
|  | |  |  |  |
| n included in fully adjusted model | | **8,614 (10,243)** | **7,502 (9,979)** | **4,875 (8,970)** |
| Friend contact | Hazard ratio for dementia per standard deviation higher social contact | 0.95 (0.85, 1.06)  p=0.36 | 0.89 (0.80, 0.99)  p=0.04 | 0.90 (0.78, 1.04)  p=0.15 |
|  | |  |  |  |
| n included in fully adjusted model | | **8,464 (10,242)** | **7,336 (9,977)** | **4,737 (8,978)** |
| Relative contact | Hazard ratio for dementia per standard deviation higher social contact | 0.90 (0.81, 1.00)  p=0.06 | 0.92 (0.82, 1.03)  p=0.13 | 0.94 (0.80, 1.10)  p=0.44 |

**Notes:** All figures adjusted for age, sex, education, social class, ethnicity, smoking, alcohol, exercise, employment status, marital status and body mass index, hypertension, diabetes mellitus and coronary heart disease at baseline age. Weighted according to inverse of probability of inclusion in fully adjusted model
