# Supplementary material for: Association of social contact with dementia and cognition: 28-year follow-up of the Whitehall II cohort study
Source: PLoS Med. 2019 Aug 2;16(8):e1002862. doi: 10.1371/journal.pmed.1002862 (PMC6677303; doi:10.1371/journal.pmed.1002862)
Supplement: S9 Table — HR, hazard ratio. (DOCX) [file pmed.1002862.s013.docx]

Supplementary table 9: Association between social contact change from age 60 to 70 years and subsequent incident dementia during mean 7.5 years follow-up: hazard ratio for dementia associated with continuous and categorical social contact change

|  | |  | **All social contact** | **Friend contact** | **Relative contact** |
| --- | --- | --- | --- | --- | --- |
| **n included in fully adjusted model (weighted n)** | | | **4,534 (8,398)** | **4,534 (8,132)** | **4,534 (8,401)** |
| **Adjusted for age, sex and baseline social contact** | *Per one-point increase in social contact score from age 60 to 70* | | 0.99 (0.93, 1.05) | 0.97 (0.89, 1.05) | 1.00 (0.93, 1.09) |
| **+ education, social class, ethnicity** |  |  | 1.02 (0.94, 1.05) | 0.98 (0.90, 1.06) | 1.01 (0.93, 1.09) |
| **+ smoking, alcohol and exercise** |  |  | 1.00 (0.94, 1.06) | 0.99 (0.91, 1.07) | 1.01 (0.93, 1.10) |
| **+ employment status** |  |  | 1.00 (0.94, 1.06) | 0.99 (0.91, 1.07) | 1.01 (0.93, 1.10) |
| **+ marital status** |  |  | 1.00 (0.94, 1.06) | 0.99 (0.91, 1.07) | 1.01 (0.93, 1.10) |
| *Categorical*  **Adjusted for age, sex, education, social class, ethnicity, smoking, alcohol, exercise, employment status and marital status** | Remain high (ref) | | 1 | 1 | 1 |
|  | Remain medium | | 1.33 (0.81, 2.19) | 1.12 (0.64, 1.96) | 0.77 (0.44, 1.37) |
|  | Remain low | | 1.18 (0.70, 1.99) | 1.22 (0.71, 2.11) | 1.12 (0.68, 1.85) |
|  | Increasing | | 1.28 (0.81, 2.02) | 1.38 (0.86, 2.20) | 1.08 (0.70, 1.67) |
|  | Decreasing | | 1.07 (0.63, 1.82) | 1.15 (0.66, 2.00) | 0.91 (0.58, 1.44) |

**Notes:** All results weighted according to inverse of probability of inclusion in fully adjusted model. Remain high = high at 60 years and 70 years; remain medium = medium at 60 years and 70 years; remain low = low at 60 years and 70 years; increasing = change from low at 60 years to medium or high at 70 years or from medium at 60 years to high at 70 years; decreasing change from high at 60 years to medium or low at 70 years or from medium at 60 years to low at 70 years
