# Supplementary material for: Association of social contact with dementia and cognition: 28-year follow-up of the Whitehall II cohort study
Source: PLoS Med. 2019 Aug 2;16(8):e1002862. doi: 10.1371/journal.pmed.1002862 (PMC6677303; doi:10.1371/journal.pmed.1002862)
Supplement: S10 Table — (DOCX) [file pmed.1002862.s014.docx]

Supplementary table 10: Description of cognitive function test scores at each study phase

| Study phase  (n) | | Reasoning  (0-65) | | Verbal memory (0-20) | | Verbal fluency  (animals) | | Verbal fluency  (s-words) | |
| --- | --- | --- | --- | --- | --- | --- | --- | --- | --- |
| 5  (7,870) | Mean (SD)  Range | 46.4 (11.3)  1, 65 | | 6.9 (2.4)  0, 18 | | 16.4 (4.2)  1, 35 | | 16.8 (4.4)  1, 35 | |
|  | *Missing* | 1,841 | 23.4 | 1,875 | 23.8 | 1,856 | 23.6 | 1,863 | 23.7 |
| 7  (6,967) | Mean (SD)  Range | 43.6 (11.3)  1, 65 | | 6.8 (2.4)  1, 17 | | 15.6 (3.9)  1, 33 | | 15.7 (4.2)  1, 35 | |
|  | *Missing* | 605 | 8.7 | 638 | 9.2 | 620 | 8.9 | 635 | 9.1 |
| 9  (6,761) | Mean (SD)  Range | 43.3 (11.3)  2, 65 | | 6.2 (2.2)  1, 16 | | 15.2 (3.8)  1, 35 | | 15.3 (4.0)  1, 35 | |
|  | *Missing* | 690 | 10.2 | 721 | 10.7 | 705 | 10.4 | 713 | 10.6 |
| 11  (6,308) | Mean (SD)  Range | 43.3 (11.4)  0, 65 | | 6.0 (2.4)  0, 20 | | 14.9 (4.0)  0, 29 | | 15.2 (4.2)  0, 35 | |
|  | *Missing* | 792 | 12.6 | 819 | 13.0 | 792 | 12.6 | 795 | 12.6 |
| 12  (5,632) | Mean (SD)  Range | 42.9 (11.2)  0, 64 | | 5.3 (2.2)  0, 18 | | 15.0 (3.9)  0, 35 | | 14.9 (4.6)  0, 35 | |
|  | *Missing* | 855 | 15.2 | 885 | 15.7 | 868 | 15.4 | 868 | 15.4 |
