# Supplementary material for: Association of social contact with dementia and cognition: 28-year follow-up of the Whitehall II cohort study
Source: PLoS Med. 2019 Aug 2;16(8):e1002862. doi: 10.1371/journal.pmed.1002862 (PMC6677303; doi:10.1371/journal.pmed.1002862)
Supplement: S11 Table — (DOCX) [file pmed.1002862.s015.docx]

Supplementary table 11: Differences in baseline cognition and cognitive change per 10 years between Whitehall II study participants with preceding medium and high social contact frequency, compared to those with low social contact

| Social domain | Cognitive domain | Social contact tertile (reference group=low) | Fully-adjusted differences | |
| --- | --- | --- | --- | --- |
|  |  |  | **Baseline cognition**  (standard deviations) | **Cognitive change**  (standard deviations / 10y) |
| **All social contact** | Combined cognition | Medium | 0.02 (-0.02, 0.06) | 0.00 (-0.02, 0.02) |
|  |  | High | **0.07 (0.03, 0.11)** | -0.01 (-0.03, 0.01) |
|  | Verbal fluency | Medium | 0.02 (-0.02, 0.07) | -0.00 (-0.03, 0.02) |
|  |  | High | **0.08 (0.03, 0.12)** | -0.00 (-0.03, 0.02) |
|  | Verbal memory | Medium | 0.03 (-0.02, 0.08) | -0.00 (-0.03, 0.03) |
|  |  | High | **0.05 (0.00, 0.10)** | -0.01 (-0.04, 0.02) |
|  | Reasoning | Medium | -0.01 (-0.05, 0.03) | 0.00 (-0.02, 0.02) |
|  |  | High | **0.01 (-0.03, 0.05)** | -0.01 (-0.03, 0.01) |
|  |  |  |  |  |
| **Friend contact** | Combined cognition | Medium | 0.01 (-0.04, 0.05) | -0.1 (-0.04, 0.00) |
|  |  | High | **0.08 (0.03, 0.12)** | **-0.03 (-0.05, -0.00)** |
|  | Verbal fluency | Medium | 0.02 (-0.03, 0.07) | -0.02 (-0.04, 0.01) |
|  |  | High | **0.10 (0.05, 0.15)** | -0.02 (-0.05, 0.00) |
|  | Verbal memory | Medium | 0.03 (-0.02, 0.08) | -0.01 (-0.04, 0.02) |
|  |  | High | **0.04 (0.01, 0.09)** | -0.02 (-0.06, 0.01) |
|  | Reasoning | Medium | 0.02 (-0.03, 0.06) | 0.00 (-0.02, 0.02) |
|  |  | High | 0.02 (-0.02, 0.06) | -0.01 (-0.03, 0.01) |
|  |  |  |  |  |
| **Relative contact** | Combined cognition | Medium | 0.02 (-0.02, 0.06) | -0.00 (-0.02, 0.02) |
|  |  | High | 0.01 (-0.03, 0.06) | 0.00 (-0.02, 0.03) |
|  | Verbal fluency | Medium | 0.01 (0.03, 0.06) | 0.01 (-0.01, 0.03) |
|  |  | High | 0.02 (-0.03, 0.07) | 0.00 (-0.03, 0.03) |
|  | Verbal memory | Medium | 0.03 (-0.01, 0.08) | -0.00 (-0.03, 0.03) |
|  |  | High | -0.01 (-0.06, 0.05) | 0.02 (-0.02, 0.05) |
|  | Reasoning | Medium | -0.00 (-0.04, 0.04) | -0.01 (-0.02, 0.01) |
|  |  | High | 0.00 (-0.04, 0.05) | -0.00 (-0.03, 0.02) |

Notes: Results adjusted for age, sex, education, social class, ethnicity, smoking, alcohol, exercise, employment status, and marital status at baseline; bold figures indicate p < 0.05
