# Supplementary material for: Association of social contact with dementia and cognition: 28-year follow-up of the Whitehall II cohort study
Source: PLoS Med. 2019 Aug 2;16(8):e1002862. doi: 10.1371/journal.pmed.1002862 (PMC6677303; doi:10.1371/journal.pmed.1002862)
Supplement: S12 Table — (DOCX) [file pmed.1002862.s016.docx]

Supplementary table 12: Differences in baseline cognition and cognitive change per 10 years between Whitehall II participants with preceding high and low social contact frequency, according to whether subsequently developed dementia

| Social domain | Cognitive domain | Dementia-free | |  | Dementia cases | |
| --- | --- | --- | --- | --- | --- | --- |
|  |  | **Baseline cognition**  (standard deviations) | **Cognitive change**  (standard deviations / 10y) |  | **Baseline cognition**  (standard deviations) | **Cognitive change**  (standard deviations / 10y) |
| **All social contact** | Combined cognition | **0.06 (0.02, 0.10)** | -0.01 (-0.03, 0.00) |  | **0.42 (0.06, 0.75)** | -0.16 (-0.38, 0.06) |
|  | Verbal fluency | **0.07 (0.03, 0.12)** | -0.01 (-0.03, 0.02) |  | 0.22 (-0.10, 0.54) | -0.07 (-0.29, 0.14) |
|  | Verbal memory | 0.04 (-0.01, 0.09) | -0.01 (-0.04, 0.02) |  | **0.51 (0.14, 0.88)** | -0.20 (-0.46, 0.05) |
|  | Reasoning | 0.01 (-0.03, 0.05) | -0.01 (-0.03, 0.01) |  | 0.09 (-0.20, 0.38) | 0.00 (-0.21, 0.21) |
|  |  |  |  |  |  |  |
| **Friend contact** | Combined cognition | **0.08 (0.03, 0.12)** | **-0.03 (-0.05, -0.00)** |  | **0.35 (0.01, 0.69)** | **-0.28 (-0.50, -0.06)** |
|  |  |  |  |  |  |  |
| **Relative contact** | Combined cognition | -0.01 (-0.05, 0.04) | 0.00 (-0.02, 0.03) |  | 0.28 (-0.08, 0.63) | -0.03 (-0.26, 0.21) |

Notes: Baseline cognition centred at age 56 years; Number included in analysis of dementia-free participants = 6,810, dementia-cases = 282; All figures adjusted for age, sex, education, social class, ethnicity, smoking, alcohol, exercise, employment status, and marital status at baseline; Bold figures indicate p < 0.05
